# Effect of Age on Breast Cancer Patient Prognoses: A Population-Based Study Using the SEER 18 Database

**DOI:** 10.1371/journal.pone.0165409

**Published:** 2016-10-31

**Authors:** Hai-long Chen, Mei-qi Zhou, Wei Tian, Ke-xin Meng, Hai-fei He

**Affiliations:** 1 Department of General Surgery, Zhejiang Provincial People's Hospital, Hangzhou, Zhejiang Province, China; 2 Department of Surgical Oncology, The Second Affiliated Hospital of Zhejiang University School of Medicine, Hangzhou, Zhejiang Province, China; University of North Carolina at Chapel Hill School of Medicine, UNITED STATES

## Abstract

**Background:**

Age is an important risk factor for breast cancer, but data regarding whether patient age at diagnosis is related to breast cancer survival are conflicting. This population-based study evaluated the effect of age on breast cancer prognosis and identified outcome-related factors.

**Patients and Methods:**

We searched the Surveillance, Epidemiology, and End Results (SEER) database and enrolled female primary non-metastatic cases. Patients were subdivided into seven groups, and analyses of the associations between age and overall survival (OS) and breast cancer-specific survival (BCSS) were carried out using the Kaplan-Meier method and Cox regression model, respectively. We also assessed differences in survival among three specific age groups, using the ages of 30 and 50 years as cut-offs. Stratified analyses regarding race, histology, grade, stage and hormone receptor status were also carried out.

**Results:**

A total of 133,057 female patients diagnosed with breast cancer from 2004 to 2008 were included in the current study (6.4% <40 years), Women aged 40 to 49 years and 60 to 69 years exhibited significantly better OS and BCSS, respectively (log-rank, p<0.001), than their counterparts in other groups. Middle-aged women exhibited distinctly better OS (log-rank, p<0.001) and BCSS (log-rank, p<0.001) than their counterparts in the other two age groups. Following adjustments for potential confounding factors, middle-age at breast cancer diagnosis was shown to be an independent predictor of favourable outcomes in terms of OS, but not BCSS (for OS, HR, 0.92; 95%CI, 0.87–0.98; p = 0.007; for BCSS, HR, 0.94; 95%CI, 0.80–1.01; p = 0.075, using the young group as reference). Stratified analysis showed that middle-age was significantly associated with increased survival, except among patients with stage III disease, and that elderly women faced worse prognoses than younger patients.

**Conclusion:**

Our results indicate that younger breast cancer patients exhibit more aggressive disease than older patients. Middle-aged patients exhibit better OS and BCSS than young and elderly patients but exhibit BCSS rates similar to those of young patients after adjustments for confounders. Stratified analysis demonstrated that middle-aged patients exhibited better survival than young patients, with the exception of patients with stage III disease. An age of 60 years or more was a significant independent predictor of a poor prognosis.

## Introduction

Breast cancer is the most common cancer among women and the leading cause of cancer-related deaths among women worldwide[1] and represents an important public health threat. Age is an important risk factor for breast cancer, as women over 50 years of age accounted for approximately 78% of new breast cancer cases and 87% of breast cancer-related deaths in 2011 in the United States[2]; however, the worldwide incidence of breast cancer among younger women has increased[3, 4] such that breast cancer is the most frequently diagnosed form of cancer among women aged <40 years[5]. Therefore, it is very important to understand the association between age at diagnosis and breast cancer survival.

It has been suggested that age at diagnosis is related to breast cancer survival, but the data regarding this issue are conflicting[6–9]. Most of the currently available data indicate that young age is associated with a poor prognosis due to the presence more invasive disease among this population[6, 7, 10–13], which is supported by other studies[8, 9, 14], although some studies have noted that elderly women experience poorer outcomes than younger patients[15, 16].

Upon reviewing these studies, we found that most used different cut-offs for age and age ranges and that most featured small datasets, which may explain the conflicting results obtained by these investigations. Thus, the relationship between age and breast cancer prognosis remains unclear and controversial. Therefore, it is necessary to elucidate the relationship between these variables in a larger population. In this study, population-based data from the National Cancer Institute’s Surveillance, Epidemiology and End Results (SEER) program were used to evaluate the effects of age on breast cancer prognosis.

## Patients and Methods

### Patients

Case lists were generated using SEER*Stat version 8.2.2. The current SEER database includes 18 population-based cancer registries representing approximately 28% of the United States population. The SEER data are publicly available for studies of cancer-related epidemiology. Data pertaining to 133,057 patients who were diagnosed with breast cancer from 2004 to 2008 were extracted from the SEER database. Data pertaining to the following types of patients were eligible for inclusion in this study: patients of female gender, patients with no other cancer diagnoses, patients with pathologically confirmed infiltrating duct carcinoma (ICD-O-3 8500/3) or lobular carcinoma (ICD-O-3 8520/3), patients with unilateral cancer, patients with histological grade I, II or III disease and patients with AJCC stage I, II or III disease. We excluded patients with inflammatory breast cancer, in situ disease, or histological grade IV (SEER program code: undifferentiated or anaplastic) disease. We calculated follow-up durations from January 1, 2004 to December 31, 2013.

### Statistical analyses

To determine the relationship between age at diagnosis and breast cancer survival, we classified age as a categorical variable and organized patients into the following seven groups: younger than 30 years (<30), 30–39 years, 40–49 years, 50–59 years, 60–69 years, 70–79 years, and older than 80 years (≥80). Patients aged 50–59 years were used as a reference,

Patient demographic characteristics and clinical features were compared among the seven age groups using a chi-squared test. The Kaplan-Meier method was used to generate survival curves, and the log-rank test was performed to compare unadjusted BCSS and OS rates among the different patient age groups. Univariate and multivariate analyses were carried out using Cox proportional hazards regression to identify outcome-related factors. BCSS was defined as the time from the date of diagnosis to the date of death from breast cancer. OS was defined as the time from the date of diagnosis to the date of death due to any cause or the last follow-up. In addition, we divided patients into the following three groups: a young group (<30 years), a middle-age group (40–60 years) and an elderly group (≥60). The Kaplan-Meier method and a log-rank test were used to calculate survival curves. HRs and 95% confidence intervals (CIs) were estimated by Cox regression analysis. All statistical analyses were performed using SPSS version 16.0 software(Chicago, IL, USA). All P values were two-sided, and P<0.05 was considered statistically significant.

## Results

### Patient demographic characteristics and clinical features

**Fig 1:**
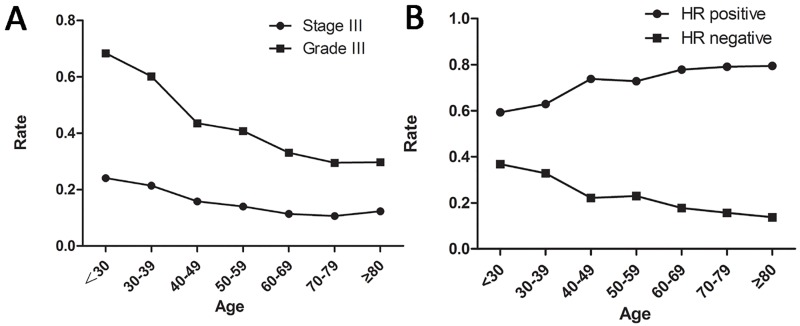
Distributions of grades, TNM stages and hormone receptor (HR) statuses among age groups. A. Distributions of grades and TNM stages. B. Distributions of hormone receptor (HR) statuses.

We enrolled a total of 133,057 patients who matched the inclusion criteria listed in the PATIENTS and METHODS. The demographic and clinical features of these patients are shown in Table 1. Patient follow-up durations ranged from 60.43 to 81.01 months. Women 40 years or younger represented only 6.4% of all enrolled patients. The 50-59-year age group comprised the most patients among the seven groups (n = 35083, 26.4%), and the <30-year age group comprised the fewest patients (n = 805, 0.61%), irrespective of race. Younger patients were more likely to have grade III and stage III disease than elderly patients (P<0.001). Furthermore, younger patients were also more likely to have hormone receptor-negative disease and less likely to have hormone receptor-positive disease than elderly patients (Fig 1).

**Table 1:** Patient demographic characteristics and clinical features.

Characteristics	<30	30–39	40–49	50–59	60–69	70–79	≥80	P^b^
	n = 805	n = 7713	n = 27829	n = 35083	n = 29480	n = 20175	n = 11972	
	No.	No.	No.	No.	No.	No.	No.	
**Mean follow-up duration (months)**	76.49	78.68	80.99	81.01	79.83	75.39	60.43	
**Race**								**<0.001**
**White**	559	5733	21578	28068	24689	17227	10609	
**Black**	153	1089	3336	3855	2606	1655	822	
**Other** ^a^	93	891	2915	3160	2185	1293	541	
**Laterality**								0.582
**Left**	412	3872	14057	17823	15010	10323	6148	
**Right**	393	3841	13772	17260	14470	9852	5824	
**Histological type**								**<0.001**
**Infiltrating duct carcinoma**	799	7507	26040	32434	26826	18127	10551	
**Lobular carcinoma**	6	207	1789	2649	2654	2048	1421	
**Histological grade**								**<0.001**
**I**	32	525	4468	6786	6603	4859	2745	
**II**	222	2546	11254	13993	13113	9363	5673	
**III**	551	4642	12107	14304	9764	5953	3554	
**7**^**th**^ **AJCC TNM stage**								**<0.001**
**I**	217	2533	12619	17940	17011	11985	6325	
**II**	394	3533	10814	12222	9105	6060	4177	
**III**	194	1647	4396	4921	3364	2130	1470	
**Hormone receptor status**								**<0.001**
**Positive**	477	4854	20538	25540	22959	15957	9516	
**Negative**	296	2532	6156	8075	5253	3146	1646	
**Borderline or unknown**	32	327	1135	1468	1268	1072	810	
**Surgery**								**<0.001**
**Breast-conserving surgery**	321	3326	15188	21493	10297	12339	6533	
**Mastectomy**	463	4240	12220	13068	18789	7514	4913	
**No surgery or unknown**	21	147	421	522	394	322	526	
**Radiation**								**<0.001**
**Yes**	422	4015	15420	20466	17207	10314	3819	
**No**	360	3435	11627	13659	11517	9417	7940	
**Unknown**	23	263	782	958	756	444	213	

^a^ Other includes American Indians/Alaska natives and Asians/Pacific Islanders.

^b^P values were calculated among all groups using a chi-squared test, and bold type indicates significance.

### Survival analysis

**Fig 2:**
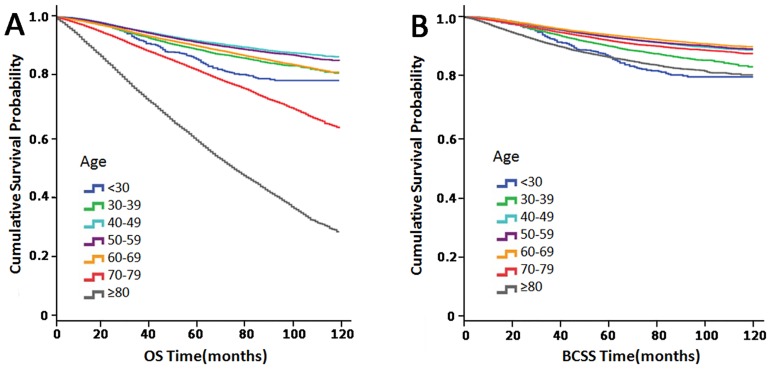
Kaplan-Meier plot comparing (A) overall survival (OS) and (B) breast cancer-specific survival (BCSS) among the seven age groups.

Kaplan-Meier plots were generated to compare OS and BCSS among the different age groups, and the results are presented in Fig 2. Significant differences in survival were observed among the groups. The analysis showed that age was associated with OS and BCSS (P<0.001). Patients aged 40–49 and 50–59 years faced better prognoses than patients in other age groups. Patients aged <30 and 30–39 years exhibited worse OS and BCSS than patients aged 40–49 and 50–59 years and slightly better OS than patients aged 70–79 and >80 years. However, patients aged 60–69 years exhibited the best BCSS among all groups.

### Analyses of survival-related factors using Cox proportional hazard regression models

The results of the BCSS and OS analyses, which were performed using univariate and multivariate Cox proportional hazard regression models, are shown in S1 and S2 Tables and Table 2. According to the results of the multivariate analysis, as shown in S2 Table, patients aged 40–49 years exhibited significantly better OS (HR, 0.87; 95%CI, 0.83 to 0.91, P<0.001) and BCSS (HR, 0.93; 95%CI, 0.88 to 0.98, P = 0.006) than patients aged 50–59 years. In addition, patients aged 30–39 years exhibited OS (HR, 0.98; 95%CI, 0.92 to 1.05, P = 0.572) and BCSS (HR, 1.00; 95%CI, 0.93 to 1.07, P = 0.939) rates similar to those of patients aged 50–59 years. However, patients in other age groups exhibited significantly worse prognoses, regardless of OS and BCSS. In addition, we found that black breast cancer patients exhibited shorter OS and BCSS than patients of other races and that American Indians/Alaskan natives and Asians/Pacific Islanders faced the best prognoses, indicating that breast cancer prognosis is affected by race.

**Table 2:** Analysis of overall survival and breast cancer-specific survival across the different age groups.

Age(years)	OS			BCSS		
	Events/Patients(%)	HR(95%CI) ^a^	P	Events/Patients(%)	HR(95%CI) ^a^	P
**<30**	153/805(19.0)	**1.19(1.01–1.39)**	**0.039**	143/805(17.8)	**1.20(1.02–1.42)**	**0.032**
**30–39**	1110/7713(14.4)	0.98(0.92–1.05)	0.532	981/7713(12.7)	1.00(0.93–1.07)	0.939
**40–49**	2934/27829(10.5)	**0.87(0.83–0.91)**	**<0.001**	2452/27829(8.8)	**0.93(0.88–0.98)**	**0.006**
**50–59**	3984/35083(11.4)	1		3015/35083(8.6)	1	
**60–69**	4069/29480(13.8)	**1.40(1.34–1.47)**	**<0.001**	2279/29480(7.7)	**1.12(1.06–1.18)**	**<0.001**
**70–79**	5371/20175(26.6)	**3.01(2.89–3.14)**	**<0.001**	1902/20175(9.4)	**1.59(1.50–1.68)**	**<0.001**
**≥80**	6798/11972(56.8)	**7.66(7.36–7.97)**	**<0.001**	1627/11972(13.6)	**2.71(2.55–2.89)**	**<0.001**

^a^Multivariate Cox regression, adjusted for race, histologic type, grade, TNM stage, hormone receptor status, surgical treatment received, and radiation treatment received.

HR, hazard ratio; CI, confidence interval.

In addition, lower histological grade, lower TNM stage, positive hormone receptor status, breast-conserving surgery and radiation therapy were independently associated with better BCSS and OS, according to the results of our univariate and multivariate analyses.

### Survival comparisons among the three groups

**Fig 3:**
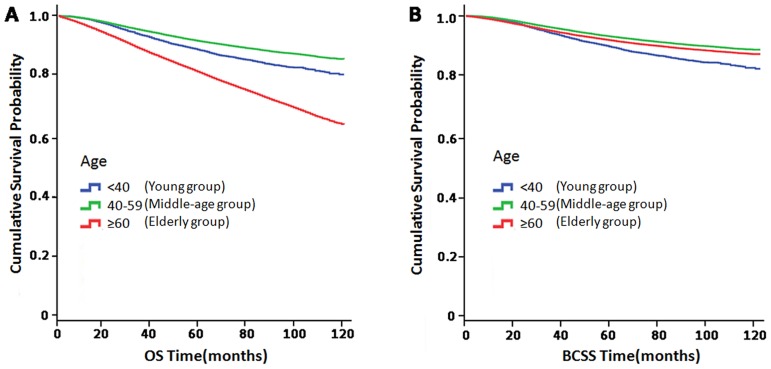
Kaplan-Meier plot comparing (A) overall survival (OS) and (B) breast cancer-specific survival (BCSS) among the three age groups (young group, middle-age group and elderly group).

Based on the aforementioned results, we divided patients into the following three groups: a young group (<40 years), a middle-age group (40–59 years), and an elderly group (≥60 years), to investigate the correlation between survival and age. Kaplan-Meier plots showed that the middle-aged group exhibited significantly better OS and BCSS than the young and elderly groups (P<0.001). Young patients exhibited better OS and worse BCSS than elderly patients (Fig 3).

A multivariate Cox proportional hazard model was used to correct for confounders (Table 3). We observed that middle age at breast cancer diagnosis was independently predictive of better OS, but not BCSS (for OS, HR, 0.92; 95%CI, 0.87–0.98; p = 0.007; for BCSS, HR, 0.94; 95%CI, 0.80–1.01; p = 0.075, using the young group as a reference). However, an age >60 at diagnosis was an independent predictor of a poor prognosis with respect to OS and BCSS (for OS, HR, 2.77; 95%CI, 2.62–2.94; p<0.001; for BCSS, HR, 1.46; 95%CI, 1.37–1.56; p<0.001, using the young group as a reference).

**Table 3:** Multivariate Cox proportional hazard model for assessing outcome-related factors.

	OS			BCSS		
	HR	95%CI	P^b^	HR	95%CI	P^b^
**Age (years)**						
**<40**	1			1		
**40–59**	0.92	0.87–0.98	**0.007**	0.94	0.80–1.01	0.075
**≥60**	2.77	2.62–2.94	**<0.001**	1.46	1.37–1.56	**<0.001**
**Race**						
**White**	1			1		
**Black**	1.35	1.30–1.40	**<0.001**	1.38	1.31–1.44	**<0.001**
**Other** ^a^	0.74	0.70–0.78	**<0.001**	0.80	0.75–0.86	**<0.001**
**Histological type**						
**Infiltrating duct carcinoma**	1			1		
**Lobular carcinoma**	0.97	0.93–1.02	0.265	1.03	0.96–1.11	0.368
**Histological grade**						
**I**	1			1		
**II**	1.19	1.14–1.24	**<0.001**	2.16	1.97–2.36	**<0.001**
**III**	1.54	1.48–1.61	**<0.001**	3.54	3.23–3.87	**<0.001**
**7**^**th**^ **AJCC TNM stage**						
**I**	1			1		
**II**	1.73	1.67–1.78	**<0.001**	3.01	2.85–3.18	**<0.001**
**III**	4.08	3.94–4.23	**<0.001**	8.57	8.10–9.07	**<0.001**
**Hormone receptor status**						
**Positive**	1			1		
**Negative**	1.42	1.38–1.47	**<0.001**	1.76	1.69–1.83	**<0.001**
**Borderline or unknown**	1.27	1.20–1.33	**<0.001**	1.34	1.24–1.46	**<0.001**
**Surgery**						
**Breast-conserving surgery**	1			1		
**Mastectomy**	1.05	1.01–1.08	**0.005**	1.25	1.20–1.31	**<0.001**
**No surgery or unknown**	2.82	2.65–3.01	**<0.001**	3.71	3.42–3.02	**<0.001**
**Radiation**						
**Yes**	1			1		
**No**	1.66	1.62–1.71	**<0.001**	1.26	1.21–1.31	**<0.001**
**Unknown**	1.24	1.15–1.35	**<0.001**	1.15	1.03–1.27	**<0.001**

Abbreviation: CI, confidence interval; HR, hazard ratio.

^a^Other includes American Indians/Alaska natives and Asian/Pacific Islanders.

^b^P values were adjusted using a multivariate Cox proportional hazard regression model including all factors, and bold type indicates significance.

Stratified analysis showed that middle-aged patients of all races, infiltrating duct carcinoma and lobular carcinoma patients, grade II-III disease patients, stage I-II disease patients and hormone receptor-positive patients exhibited significantly increased survival compared with other patients (Table 4). However, middle-aged patients with stage III disease did not exhibit better survival than young patients (for OS, HR, 0.97; 95%CI, 0.89–1.07; p = 0.557; for BCSS, HR, 0.94; 95%CI, 0.85–1.03; p = 0.164). Elderly patients in almost every subgroup exhibited worse survival than young patients.

**Table 4:** Stratified analysis of the association between age at diagnosis and breast cancer patient survival.

Variable	Middle-age group				Elderly group			
	OS		BCSS		OS		BCSS	
	HR(95%CI)	P ^a^	HR(95%CI)	P ^a^	HR(95%CI)	P ^a^	HR(95%CI)	P ^a^
Race								
White	0.70(0.65–0.76)	**<0.001**	0.63(0.58–0.68)	**<0.001**	2.05(1.91–2.20)	**<0.001**	0.79(0.73–0.85)	**<0.001**
Black	0.88(0.78–1.00)	**0.043**	0.77(0.67–0.88)	**<0.001**	1.57(1.39–1.78)	**<0.001**	0.75(0.65–0.87)	**<0.001**
Other ^b^	0.71(0.59–0.87)	**0.001**	0.63(0.51–0.78)	**<0.001**	1.59(1.31–1.92)	**<0.001**	0.74(0.59–0.92)	**0.007**
Histological type								
Infiltrating duct carcinoma	0.73(0.67–0.77)	**<0.001**	0.65(0.61–0.70)	**<0.001**	1.86(1.75–1.97)	**<0.001**	0.75(0.70–0.80)	**<0.001**
Lobular carcinoma	0.65(0.44–0.96)	**0.03**	0.53(0.35–0.80)	**0.002**	2.35(1.61–3.44)	**<0.001**	0.87(0.58–1.30)	0.501
Histological grade								
I	0.96(0.61–1.52)	0.865	0.61(0.35–1.05)	0.073	5.99(3.82–9.41)	**<0.001**	1.11(0.65–1.89)	0.711
II	0.73(0.65–0.83)	**<0.001**	0.60(0.53–0.69)	**<0.001**	2.58(2.29–2.91)	**<0.001**	0.85(0.74–0.96)	**0.012**
III	0.93(0.87–1.00)	**0.035**	0.88(0.81–0.94)	**0.001**	2.04(1.91–2.18)	**<0.001**	1.16(1.08–1.25)	**<0.001**
AJCC stage (7th ed)								
I	0.73(0.62–0.86)	**<0.001**	0.52(0.43–0.63)	**<0.001**	3.47(2.96–4.07)	**<0.001**	0.67(0.56–0.81)	**<0.001**
II	0.87(0.79–0.95)	**0.003**	0.78(0.71–0.86)	**<0.001**	2.53(2.32–2.77)	**<0.001**	1.13(1.03–1.25)	**0.014**
III	0.97(0.89–1.07)	0.557	0.94(0.85–1.03)	0.164	2.00(1.83–2.19)	**<0.001**	1.38(1.26–1.52)	**<0.001**
Hormone receptor status								
Positive	0.68(0.62–0.74)	**<0.001**	0.57(0.52–0.63)	**<0.001**	2.21(2.04–2.40)	**<0.001**	0.75(0.68–0.82)	**<0.001**
Negative	0.95(0.87–1.04)	0.268	0.90(0.82–0.99)	**0.035**	1.79(1.64–1.96)	**<0.001**	1.10(1.00–1.21)	**0.049**
Borderline or unknown	0.78(0.58–1.03)	0.078	0.67(0.49–0.90)	**0.009**	2.55(1.95–3.33)	**<0.001**	0.95(0.71–1.28)	0.756

HR, hazard ratio; CI, confidence interval.

^a^Univariate Cox regression, using the young group as a reference, and bold type indicates significance.

^b^Including American Indians, Alaska natives, Asians, Pacific Islanders and Unknown.

## Discussion

In this study, we attempted to determine the effect of age on survival among breast cancer patients and evaluated a total of 133,057 patients for this purpose. We chose the period from 2004 to 2008 to ensure adequate follow-up. The 50-59-year age group was the largest group of patients enrolled in this study (26.4%), while the <30-year age group was the smallest group of patients enrolled in this study. Patients aged <40 represented only a very small proportion (6.4%) of our study population. These percentages were consistent with those of a large retrospective cohort involving 243,012 breast cancer patients who were diagnosed between 1988 and 2003 in the United States, as 6.4% patients in this cohort were also younger than 40 years of age[17]. Patients ≥40 years constituted the majority of patients (93.6%) in our cohort, indicating that women aged 40 years or older have a higher risk of developing breast cancer than younger women. Therefore, routine breast cancer screenings for women ≥40 years are necessary.

In this study, we divided patients into seven groups according to age. Kaplan-Meier survival analysis showed that patients aged 40–49 years exhibited significantly better OS than patients in other groups, followed by patients aged 50–59 years, and that women aged 70 years or more exhibited significantly poorer survival than patients in other groups. However, patients aged 60–69 years exhibited better BCSS than patients aged 40–49 and 50–59 years, while patients aged >80 years, who exhibited the worst OS, exhibited BCSS rates similar to those of patients aged <30 years. When we divided patients into three groups, we noted that middle-aged women exhibited significantly better OS and BCSS than patients in other groups, while elderly women exhibited worse OS but better BCSS than young women. These results may reflect the higher frequencies of comorbidities noted among elderly patients, as these patients exhibited a significantly lower breast cancer-specific mortality (BCSM) rate than young women.

As mentioned above, the majority of previous studies have reported that young age is associated with a poor prognosis among breast cancer patients, but this issue remains controversial, as the results of studies performed in Iran[14], Nigeria[18], Egypt[8], and even the United States[19] do not support this conclusion. However, most of these studies allocated patients into two groups and used age cut-offs of 35 or 40 years. As a result, the age ranges of the older groups in these studies were extremely large and included middle-aged and senile patients, which may have affected the results of these studies. A few studies performed comprehensive analyses involving patients of all ages. Balabram et al[20] performed a retrospective cohort study of 767 breast cancer patients in Brazil, the results of which indicated that women aged ≥70 and ≤35 exhibited shorter cancer-specific survival than patients aged between 36 and 69 years. Roder et al[21] analysed 493 breast cancer patients diagnosed from 1998–2005 in Australia and found that women under 40 years and over 70 years exhibited poorer overall survival than women between 40 and 69 years. Similarly, Brandt et al[12] studied 4,453 women who were diagnosed with breast cancer between 1961 and 1991 at a single institution in Sweden and were followed up for 10 years regarding breast cancer-specific mortality. They found that women under 40 and above 80 years of age had poorer prognoses than women in other age groups. These results were generally consistent with ours.

The proportions of patients with grade III and stage III disease decreased with age, and younger patients were more likely to be diagnosed with a higher grade and a more advanced stage of disease. This may be due to poor breast cancer screening in young women, as the incidence of the disease in this population is low, which results in patients having larger masses and more advanced disease when they are diagnosed. Additionally, younger patients were more likely to be hormone receptor-negative, and elderly patients were more likely to be hormone receptor-positive, findings consistent with those of many studies[4, 6, 8, 9, 22]. In most studies, factors such as disease stage, grade, and hormone receptor status were classical predictors of breast cancer survival[5, 23, 24]. The univariate and multivariate analyses performed in this study showed that white race, higher grade, advanced stage, and hormone receptor positivity were significantly associated with poor OS and BCSS.

Additionally, the presence of these characteristics in younger patients was associated with more aggressive disease and a poorer prognosis. However, these unfavourable characteristics were not the only factors associated with poor patient prognoses. Following adjustments for race, histologic type, grade, TNM stage, hormone receptor status, surgery and radiation therapy, old age was shown to be an independent risk factor for poor OS and BCSS, and middle age was independently associated with better OS and BCSS. Young patients exhibited shorter OS than middle-aged women, but no significant difference was noted between these groups with respect to BCSS. Overall, middle-aged women exhibited longer survival times, most likely as a result of early disease detection. Additionally, these patients exhibited less aggressive disease and better therapy tolerance. In contrast, young women were diagnosed relatively late in their disease courses, and elderly women were unable to tolerate certain types of adjuvant therapy due to comorbidities, resulting in worse prognoses.

In addition, previous studies also determined that young patients were more likely to have HER2-positive disease[4, 22]. However, HER2 status was not documented in the SEER database before 2010; therefore, we did not include this variable in our analysis, but it should be included in future analyses.

Stratified analysis showed that middle-aged patients of all races, infiltrating duct carcinoma and lobular carcinoma patients, grade II-III disease patients, stage I-II disease patients and hormone receptor-positive patients exhibited significantly better survival than patients in other groups, whereas elderly patients in almost all subgroups exhibited worse survival than young patients. Significant differences were not observed between young patients and middle-aged patients with stage II breast cancer.

This study was not without limitations, as we analysed only patients who were treated with surgery and radiation because the public SEER database does not contain information regarding adjuvant chemotherapy or endocrine therapy. This may have affected our results. Moreover, the SEER database does not contain information regarding patient socioeconomic status, which may have affected patient treatment plans.

## Conclusion

Our results indicate that younger breast cancer patients exhibited more aggressive disease than older patients. Middle-aged patients exhibited distinctly better OS and BCSS than young patients and elderly patients but exhibited a BCSS rate similar to that of young patients after adjustments for confounders. Stratified analysis demonstrated that middle-aged patients exhibited better survival than young patients, with the exception of patients with stage III disease. However, an age of 60 years or more was a significant independent predictor of a poor prognosis.

## Supporting Information

S1 Table Univariate Cox proportional hazard model for assessing outcome-related factors. (DOCX)

S2 Table Multivariate Cox proportional hazard model for assessing outcome-related factors. (DOCX)
