# Supplementary material for: Effect of Age on Breast Cancer Patient Prognoses: A Population-Based Study Using the SEER 18 Database
Source: PLoS One. 2016 Oct 31;11(10):e0165409. doi: 10.1371/journal.pone.0165409 (PMC5087840; doi:10.1371/journal.pone.0165409)
Supplement: S1 Table — (DOCX) [file pone.0165409.s001.docx]

**S1 Table. Univariate Cox proportional hazard model for assessing outcome-related factors**

|  | **OS** | | |  | **BCSS** | | |
| --- | --- | --- | --- | --- | --- | --- | --- |
|  | **HR** | **95% CI** | **P** |  | **HR** | **95% CI** | **P** |
| **Age(years)** |  |  |  |  |  |  |  |
| **<30** | 1.78 | 1.51-2.09 | **<0.001** |  | 2.18 | 1.84-2.58 | **<0.001** |
| **30-39** | 1.31 | 1.22-1.40 | **<0.001** |  | 1.52 | 1.42-1.64 | **<0.001** |
| **40-49** | 0.93 | 0.89-0.97 | **0.002** |  | 1.03 | 0.97-1.08 | 0.34 |
| **50-59** | 1 |  |  |  | 1 |  |  |
| **60-69** | 1.23 | 1.18-1.29 | **<0.001** |  | 0.91 | 0.86-0.96 | **0.001** |
| **70-79** | 2.53 | 2.43-2.64 | **<0.001** |  | 1.17 | 1.11-1.24 | **<0.001** |
| **≥80** | 6.83 | 6.56-7.10 | **<0.001** |  | 2.09 | 1.96-2.22 | **<0.001** |
|  |  |  |  |  |  |  |  |
| **Race** |  |  |  |  |  |  |  |
| **White** | 1 |  |  |  | 1 |  |  |
| **Black** | 1.58 | 1.52-1.64 | **<0.001** |  | 2.00 | 1.91-2.10 | **<0.001** |
| **Other** ^a^ | 0.7 | 0.67-0.74 | **<0.001** |  | 0.86 | 0.80-0.92 | **<0.001** |
|  |  |  |  |  |  |  |  |
| **Histological type** |  |  |  |  |  |  |  |
| **Infiltrating duct carcinoma** | 1 |  |  |  | 1 |  |  |
| **Lobular carcinoma** | 1.04 | 0.99-1.09 | 0.095 |  | 0.86 | 0.80-0.92 | **<0.001** |
|  |  |  |  |  |  |  |  |
| **Histological grade** |  |  |  |  |  |  |  |
| **I** | 1 |  |  |  | 1 |  |  |
| **II** | 1.43 | 1.37-1.49 | **<0.001** |  | 3.21 | 2.93-3.51 | **<0.001** |
| **III** | 2.25 | 2.16-2.34 | **<0.001** |  | 8.24 | 7.56-8.98 | **<0.001** |
|  |  |  |  |  |  |  |  |
| **7^th^ TNM AJCC stage** |  |  |  |  |  |  |  |
| **I** | 1 |  |  |  | 1 |  |  |
| **II** | 1.9 | 1.84-1.96 | **<0.001** |  | 4.12 | 3.91-4.34 | **<0.001** |
| **III** | 4.21 | 4.08-4.35 | **<0.001** |  | 12.51 | 11.88-13.18 | **<0.001** |
|  |  |  |  |  |  |  |  |
| **Hormonal-receptor Status** |  |  |  |  |  |  |  |
| **Positive** | 1 |  |  |  | 1 |  |  |
| **Negative** | 1.77 | 1.72-1.82 | **<0.001** |  | 2.92 | 2.82-3.03 | **<0.001** |
| **Borderline or unknown** | 1.59 | 1.50-1.67 | **<0.001** |  | 1.7 | 1.58-1.84 | **<0.001** |
|  |  |  |  |  |  |  |  |
| **Surgery** |  |  |  |  |  |  |  |
| **Breast-conserving surgery** | 1 |  |  |  | 1 |  |  |
| **Mastectomy** | 1.83 | 1.78-1.88 | **<0.001** |  | 2.51 | 2.41-2.60 | **<0.001** |
| **No surgery or Unknown** | 6.32 | 5.96-6.70 | **<0.001** |  | 9.34 | 8.67-10.07 | **<0.001** |
|  |  |  |  |  |  |  |  |
| **Radiation** |  |  |  |  |  |  |  |
| **Yes** | 1 |  |  |  | 1 |  |  |
| **No** | 1.88 | 1.83-1.93 | **<0.001** |  | 1.45 | 1.40-1.51 | **<0.001** |
| **Unknown** | 1.45 | 1.34-1.57 | **<0.001** |  | 1.49 | 1.34-1.65 | **<0.001** |

Abbreviation: CI, confidence interval; HR, hazard ratio

^a^ Other includes American Indian/native Alaskan and Asian/Pacific Islander.

^b^ Univariate Cox regression
