# Supplementary material for: Effect of Age on Breast Cancer Patient Prognoses: A Population-Based Study Using the SEER 18 Database
Source: PLoS One. 2016 Oct 31;11(10):e0165409. doi: 10.1371/journal.pone.0165409 (PMC5087840; doi:10.1371/journal.pone.0165409)
Supplement: S2 Table — (DOCX) [file pone.0165409.s002.docx]

**S2 Table.** **Multivariate Cox proportional hazard model for assessing outcome-related factors**

|  | **OS** | | |  | **BCSS** | | |
| --- | --- | --- | --- | --- | --- | --- | --- |
|  | **HR** | **95% CI** | **P** |  | **HR** | **95% CI** | **P** |
| **Age(years)** |  |  |  |  |  |  |  |
| **<30** | 1.19 | 1.01-1.39 | **0.039** |  | 1.20 | 1.02-1.42 | **0.032** |
| **30-39** | 0.98 | 0.92-1.05 | 0.572 |  | 1.00 | 0.93-1.07 | 0.939 |
| **40-49** | 0.87 | 0.83-0.91 | **<0.001** |  | 0.93 | 0.88-0.98 | **0.006** |
| **50-59** | 1 | 1 |  |  | 1 | 1 |  |
| **60-69** | 1.40 | 1.34-1.47 | **<0.001** |  | 1.12 | 1.06-1.18 | **<0.001** |
| **70-79** | 3.01 | 2.89-3.14 | **<0.001** |  | 1.59 | 1.50-1.68 | **<0.001** |
| **≥80** | 7.66 | 7.36-7.97 | **<0.001** |  | 2.71 | 2.55-2.89 | **<0.001** |
|  |  |  |  |  |  |  |  |
| **Race** |  |  |  |  |  |  |  |
| **White** | 1 |  |  |  | 1 |  |  |
| **Black** | 1.45 | 1.39-1.50 | **<0.001** |  | 1.41 | 1.35-1.48 | **<0.001** |
| **Other** | 0.80 | 0.76-0.85 | **<0.001** |  | 0.83 | 0.77-0.89 | **<0.001** |
|  |  |  |  |  |  |  |  |
| **Histological type** |  |  |  |  |  |  |  |
| **Infiltrating duct carcinoma** | 1 |  |  |  | 1 |  |  |
| **Lobular carcinoma** | 0.92 | 0.88-0.97 | **<0.001** |  | 1.01 | 0.94-1.09 | 0.724 |
|  |  |  |  |  |  |  |  |
| **Histological grade** |  |  |  |  |  |  |  |
| **I** | 1 |  |  |  | 1 |  |  |
| **II** | 1.20 | 1.15-1.25 | **<0.001** |  | 2.17 | 1.99-2.38 | **<0.001** |
| **III** | 1.62 | 1.55-1.69 | **<0.001** |  | 3.61 | 3.29-3.95 | **<0.001** |
|  |  |  |  |  |  |  |  |
| **7^th^ TNM AJCC stage** |  |  |  |  |  |  |  |
| **I** | 1 |  |  |  | 1 |  |  |
| **II** | 1.73 | 1.67-1.78 | **<0.001** |  | 3.01 | 2.85-3.17 | **<0.001** |
| **III** | 4.13 | 3.98-4.28 | **<0.001** |  | 8.60 | 9.13-9.10 | **<0.001** |
|  |  |  |  |  |  |  |  |
| **Hormonal-receptor Status** |  |  |  |  |  |  |  |
| **Positive** | 1 |  |  |  | 1 |  |  |
| **Negative** | 1.50 | 1.45-1.54 | **<0.001** |  | 1.79 | 1.72-1.87 | **<0.001** |
| **Borderline or unknown** | 1.23 | 1.17-1.30 | **<0.001** |  | 1.33 | 1.23-1.44 | **<0.001** |
|  |  |  |  |  |  |  |  |
| **Surgery** |  |  |  |  |  |  |  |
| **Breast-conserving surgery** | 1 |  |  |  | 1 |  |  |
| **Mastectomy** | 1.07 | 1.03-1.10 | **<0.001** |  | 1.25 | 1.20-1.30 | **<0.001** |
| **No surgery or Unknown** | 2.61 | 2.45-2.78 | **<0.001** |  | 3.60 | 3.33-3.91 | **<0.001** |
|  |  |  |  |  |  |  |  |
| **Radiation** |  |  |  |  |  |  |  |
| **Yes** | 1 |  |  |  | 1 |  |  |
| **No** | 1.43 | 1.39-1.47 | **<0.001** |  | 1.20 | 1.15-1.25 | **<0.001** |
| **Unknown** | 1.23 | 1.14-1.33 | **<0.001** |  | 1.15 | 1.03-1.27 | **<0.001** |

Abbreviation: CI, confidence interval; HR, hazard ratio

^a^ Other includes American Indian/native Alaskan and Asian/Pacific Islander.

^b^P values were adjusted using a multivariate Cox proportional hazard regression model including all factors, and bold type indicates significance.
